# Butanol tolerance regulated by a two-component response regulator Slr1037 in photosynthetic *Synechocystis* sp. PCC 6803

**DOI:** 10.1186/1754-6834-7-89

**Published:** 2014-06-11

**Authors:** Lei Chen, Lina Wu, Jiangxin Wang, Weiwen Zhang

**Affiliations:** 1 Laboratory of Synthetic Microbiology, School of Chemical Engineering & Technology, Tianjin University, Tianjin 300072, P.R. China; 2 Key Laboratory of Systems Bioengineering, Ministry of Education of China, Tianjin 300072, P.R. China; 3 Collaborative Innovation Center of Chemical Science and Engineering, Tianjin, P.R. China

**Keywords:** Butanol, Tolerance, Response regulator, Proteomics, *Synechocystis*

## Abstract

**Background:**

Butanol production directly from CO_2_ in photosynthetic cyanobacteria is restricted by the high toxicity of butanol to the hosts. In previous studies, we have found that a few two-component signal transduction systems (TCSTSs) were differentially regulated in *Synechocystis* sp. PCC 6803 after butanol treatment.

**Results:**

To explore regulatory mechanisms of butanol tolerance, in this work, by constructing gene knockout mutants of the butanol-responsive TCSTS genes and conducting tolerance analysis, we uncovered that an orphan *slr1037* gene encoding a novel response regulator was involved in butanol tolerance in *Synechocystis*. Interestingly, the ∆*slr1037* mutant grew similarly to the wild type under several other stress conditions tested, which suggests that its regulation on butanol tolerance is specific. Using a quantitative iTRAQ LC-MS/MS proteomics approach coupled with real-time reverse transcription PCR, we further determined the possible butanol-tolerance regulon regulated by Slr1037. The results showed that, after *slr1037* deletion, proteins involved in photosynthesis and glycolysis/gluconeogenesis of central metabolic processes, and glutaredoxin, peptide methionine sulfoxide reductase and glucosylglycerol-phosphate synthase with stress-responsive functions were down-regulated, suggesting that Slr1037 may exhibit regulation to a wide range of cellular functions in combating butanol stress.

**Conclusions:**

The study provided a proteomic description of the putative butanol-tolerance regulon regulated by the *slr1037* gene. As the first signal transduction protein identified directly related to butanol tolerance, response regulator Slr1037 could be a natural candidate for transcriptional engineering to improve butanol tolerance in *Synechocystis*.

## Background

Although currently ethanol constitutes 90% of all biofuels, biofuels offer a diverse range of promising alternatives. Other fuels with better chemical properties, such as butanol with superior chemical properties in terms of energy content, volatility, corrosiveness, and its compatibility with the existing fuel storage and distribution infrastructure [1], have been proposed as the next-generation biofuels to replace gasoline, diesel, and jet fuels. Butanol is typically produced by fermentation processes employed anaerobic Gram-positive bacteria, such as *Clostridium acetobutylicum*, through an acetone-butanol-ethanol (ABE) fermentation process [2,3]. However, the relatively slow growth rate, complicated life cycle (spore-forming), and significant production of by-products associated with the anaerobic microorganisms have together made butanol production by fermentation processes less competitive with gasoline [4,5]. To address these issues, efforts have been recently made to produce butanol in more user-friendly non-native hosts, such as *Escherichia coli* and *Lactobacillus brevis*[6-10]. More recently, photosynthetic cyanobacteria have attracted a lot of attention for their potential application as 'microbial factories’ to produce biofuels and fine chemicals, due to their natural capability to utilize solar energy and CO_2_ as the sole energy and carbon sources for growth [11]. Lan and Liao (2011) constructed a modified CoA-dependent 1-butanol production pathway in cyanobacterial *Synechococcus elongatus* PCC 7942 and achieved 1-butanol production from CO_2_ and light [12]. By substituting a bifunctional aldehyde/alcohol dehydrogenase (AdhE2) with separate butyraldehyde dehydrogenase (Bldh) and NADPH-dependent alcohol dehydrogenase (YqhD), the researchers further increased the CO_2_-derived 1-butanol production fourfold [13]. While these exciting progresses demonstrated a promising future for renewable production of butanol from CO_2_, the current production level is still very low, partially due to the toxicity of butanol towards cyanobacterial cells [14,15]. Therefore, how to increase the inherent butanol tolerance of the cyanobacterial production systems may represent an important hurdle of improving their productivity [15].

Recent genome-level studies have showed that microbes tend to employ multiple resistance mechanisms in dealing with the stress of a single biofuel product [15-17], and it is thus very challenging to achieve tolerance improvement by sequential multigene modifications. To address the issue, approaches have been proposed to focus on various regulatory systems for biofuel tolerance improvement [18]. For example, overexpression of the *spo0A* gene, encoding a regulator of stationary-phase events and required for transcription of solvent formation genes in butanol-producing *Clostridium acetobutylicum*, was shown to enhance the butanol tolerance [19]. Random mutation and expression of an exogenous global regulator *irrE* from radiation-resistant *Deinococcus radiodurans* in *E. coli* resulted in improved biofuel tolerances of *E. coli* cells from several to a hundred fold [20]. Random mutagenesis of a global transcription factor cyclic AMP receptor protein (CRP) of *E. coli* resulted in a twofold growth rate increase when grown under 1.2% 1-butanol [21]. These studies demonstrated that direct manipulation of regulatory systems could provide a useful tool in tolerance engineering. However, currently very little information is available on regulatory systems controlling biofuel tolerance in photosynthetic cyanobacteria.

Two-component signal transduction systems (TCSTSs) are the predominant signal transduction system widely distributed in bacteria [22]. Interestingly, so far only one master response regulator, Spo0A, has been found to be involved in butanol tolerance in *C. acetobutylicum*[19], and no TCSTS has been found to be involved in butanol tolerance in *E. coli* or other model species [22,23]. Considering that TCSTSs have been shown to be involved in sensing and responses to a variety of environmental stresses [22], we expect that response to various biofuels may also be controlled directly or indirectly by TCSTSs. Recently, our laboratory has been focusing on exploring tolerance mechanisms of cyanobacterium *Synechocystis* sp. PCC 6803 (hereafter *Synechocystis*) to various biofuels [24-29]. Through a quantitative iTRAQ LC-MS/MS proteomics and RNA-seq transcriptomics analyses of *Synechocystis* under butanol stress, we previously identified several proteins/genes of TCSTSs differentially regulated by butanol exposure [26,29]. In this work, by constructing the gene knockout mutant and conducting phenotypic analysis, we confirmed that a novel TCSTS response regulator Slr1037 was involved in butanol tolerance in *Synechocystis*. As the first signal transduction protein confirmed related to butanol tolerance, Slr1037 could be a potential engineering target of regulatory proteins for improving butanol tolerance in *Synechocystis*[30]. Towards this end, we further determined the possible butanol-tolerance regulon regulated by Slr1037 using a quantitative iTRAQ proteomics approach coupled with quantitative reverse-transcription PCR (RT-qPCR).

## Results and discussion

### Butanol tolerance analysis of selected TCSTS mutants

**Figure 1:**
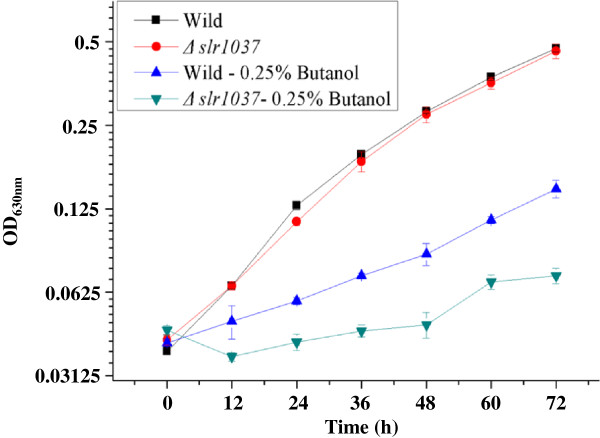
**Growth of the wild type and the mutant ∆*slr1037 *of *Synechocystis *in BG11 media with or without butanol.** BG11 medium was supplemented with 0.25% (*v*/*v*) butanol.

A recent survey showed that *Synechocystis* contains at least 91 genes encoding TCS components, including 27 histidine kinases, 20 hybrid-type histidine kinases, and 44 response regulators [31]. Our previous proteomic and transcriptomic analysis found four histidine kinases and seven response regulators differentially regulated by butanol [26,29]. In this study, two TCSTS response regulator genes, *sll1708* and *slr1037,* and one transcriptional regulator gene, *sll0690,* were selected for construction of knockout mutants and validation of their involvement in butanol tolerance. Currently, no functional information is available for *slr1037* and *sll0690*, while *sll1708* (also called Rre17 in the literature) was found to be involved in perception of salt stress and transduction of the signal in *Synechocystis*[32]. After chromosomal integration and full segregation as confirmed by PCR and sequencing (data not shown, the PCR primers for mutant construction and validation are listed in Additional file 1: Table S1.), the mutants were grown in parallel with the wild-type *Synechocystis* in both normal BG11 medium and the BG11 medium supplemented with 0.25% (*v*/*v*) butanol. Comparative analysis showed that there is no visible difference in terms of growth patterns between the wild-type strain and all three mutants in the normal BG11 medium, which was also confirmed using flow cytometric analysis where no morphologic difference was detected (data not shown); however, gene disruption of *slr1037* led to an almost twofold increase in butanol sensitivity, suggesting that the gene was involved in butanol resistance (Figure 1). The growth rates during the exponential growth phase (12 to 36 h) were determined as 0.00496, 0.00497, 0.00037, and 0.00250 OD_630_ change per hour for the wild type and the ∆*slr1037* mutant grown under normal BG11, and the wild type and the ∆*slr1037* mutant grown under 0.25% butanol, respectively. No obvious change in terms of butanol tolerance was observed for the knockout mutants of *sll0690* and *sll1708* genes (data not shown), suggesting that their differential regulation by butanol exposure revealed by previous proteomic and transcriptomic analysis may be part of the secondary responses in cells [26,29,32].

The *slr1037* gene is also annotated as a CheY subfamily Rre10 response regulator based on protein domain analysis; however, scant functional clues are provided in the literature, although previous large-scale protein-protein interaction analyses suggested that it could work together with a histidine kinase Hik1 (*slr1393*) [33,34]. Genomic context analysis showed that no histidine kinase was identified in its neighborhood, suggesting that *slr1037* is an orphan response regulator gene. In addition, two genes upstream of *slr1037*, which were transcribed in the opposite direction (*sll0985* and *sll0984*), and three genes downstream of *slr1037* (*sll0983*, *sll0982,* and *sll0981*), which were also transcribed in the opposite direction, all encode functionally unknown hypothetical genes, providing no functional clues for *slr1037*. A National Center for Biotechnology Information (NCBI) *BlastP* search using the deduced Slr1037 protein sequence showed that the genes shared 61%, 58%, 58%, 57%, and 54% identity with the response regulators from several other cyanobacteria, *Cyanothece* sp. PCC 7822, *Cyanothece* sp. ATCC 51142, *Nostoc* sp. PCC 7120, *Anabaena variabilis* ATCC 29413, and *Crocosphaera watsonii* WH 0003, respectively. However, none of the response regulators in these species was so far functionally characterized.

**Figure 2:**
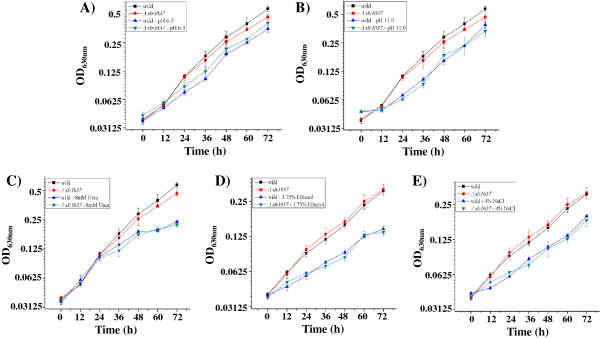
**Growth time courses of the wild type and the ∆*slr1037 *mutant of *Synechocystis *under various stress conditions. A)** pH 6.5; **B)** pH 11.0; **C)** 8 mM urea; **D)** 1.75% ethanol; **E)** 4.0% NaCl.

To determine whether the ∆*slr1037* mutant was sensitive specifically to butanol, experiments were conducted to compare growth patterns of the wild-type strain and the ∆*slr1037* mutant under several other stress conditions: 1.75% ethanol (*v*/*v*), 4% NaCl (*w*/*v*), pH 6.5, pH 11.0, and 8 mM urea. The growth experiment was repeated three times, each with three biological replicates. The results showed no visible difference between the wild type and the ∆*slr1037* mutant under the tested stress conditions (Figure 2). In addition, we calculated the growth rate for the strains under various conditions, and the results also showed no significant difference between the wild type and the ∆*slr1037* mutant under the tested stress conditions, suggesting that the *slr1037* gene may not be a master response regulator controlling general tolerance responses to multiple stresses. It is worth noting that the ∆*slr1037* mutant behaved similarly to the wild type under ethanol stress, implying some degree of the specificity of the Slr1037 mediated regulatory network against butanol.

### Overview of proteomic analysis

For the proteomic analysis, two independent cultivations for both wild-type control and the ∆*slr1037* mutant were grown in BG11 medium supplemented with 0.25% butanol, and the cells were collected by centrifugation (8,000 × *g* for 10 min at 4°C) at the exponential phase (36 h) (Figure 1), resulting two biological replicates for the wild-type and the ∆*slr1037* mutant samples. The purified protein samples were subjected to the iTRAQ LC-MS/MS proteomic analysis as described in the Methods section [26,27]. After data filtering to eliminate low-scoring spectra, the qualified spectra were matched to 1,521 proteomes, representing approximately 43% of the 3,569 predicted proteins in the *Synechocystis* genome [35]. Reproducibility of the quantitative proteomic analyses were accessed by two types of comparisons. First we labeled and mixed two biological replicates of either the wild-type or the ∆*slr1037* mutant samples directly for proteomic analysis, and the difference was plotted versus the percentage of the proteins identified. The results showed approximately 60% of the proteins with difference less than a delta error of 0.2 to 0.25, and more than 95% of the proteins with difference less than a delta error of 0.5 (Additional file 2: Figure S1A,B). Second we labeled and mixed each pair of the ∆*slr1037* mutant and the wild-type samples for proteomic analysis, and the difference between different biological pairs was plotted in Additional file 2: Figure S1C,D. The dispersion of the iTRAQ ratios of the quantified proteins was found with very similar trends between biological replicates, suggesting that the biological noise was reasonably low.

### RT-qPCR validation of proteomic data

A subset of 13 genes encoding the ∆*slr1037*-responsive proteins was randomly selected for RT-qPCR validation. Among these genes, 6 of their corresponding proteins (Slr0179, Slr0476, Slr0879, Ssr1853, Sll0830, Sll0108) were found to be up-regulated upon the *slr1037* gene deletion, and 7 (Sll0167, Slr0420, Slr1260, Sll1819, Slr0628, Sll0368, Slr1403) were down-regulated according to the proteomic data. RT-qPCR analysis showed a positive correlation between RT-qPCR and proteomic results for these genes, as 4 out of 6 up-regulated proteins have their corresponding genes up-regulated at least 1.5 fold, and 6 out of 7 down-regulated proteins have their corresponding genes down-regulated at least 1.5 fold, respectively (Table 1). The comparison suggested an overall good quality of the proteomic analysis.

**Table 1:** Comparison of ratios calculated from iTRAQ proteomics and RT-PCR analyses

**Gene ID**	Proteomic ratio*	RT-PCR ratio**	**Gene description**
slr0179	1.604 ± 0.149	1.197 ± 0.140	Hypothetical protein
slr0476	2.335 ± 0.791	1.717 ± 0.058	Hypothetical protein
slr0879	1.807 ± 0.171	1.970 ± 0.098	Conserved hypothetical protein
ssr1853	2.635 ± 1.097	1 .857 ± 0.180	Hypothetical protein
sll0830	1.643 ± 0.165	1.527 ± 0.094	Elongation factor g, putative
sll0108	2.257 ± 0.536	1.131 ± 0.152	Ammonium transporter
sll0167	-2.990 ± 1.451	1.210 ± 0.128	Hypothetical protein
slr0420	-1.838 ± 0.504	-3.448 ± 1.101	Hypothetical protein
sll1819	-1.811 ± 0.402	-3.980 ± 0.635	50S ribosomal protein L17
slr0628	-1.869 ± 0.558	-1.578 ± 0.447	30S ribosomal protein Sl4
sll0368	-1.782 ± 0.128	-2.943 ± 0.585	Pyrimidine operon regulatory protein PyrR
slr1260	-1.975 ± 0.539	-2.303 ± 0.570	Hypothetical protein
slr1403	-2.401 ± 0.295	-1.946 ± 0.243	Hypothetical protein

*Four proteomic measurements were used to calculate the average ratio and standard deviation.

**Each RT-PCR was repeated three times.

### Putative butanol-tolerance regulon mediated by Slr1037

Using a cutoff of 1.5-fold change plus a statistical significance *p*-value less than 0.05, we determined that between the ∆*slr1037* mutant and the wild type grown under 0.25% butanol, 106 and 164 unique proteins were up-regulated and down-regulated, respectively.

#### (*a*) Central metabolic processes

**Figure 3:**
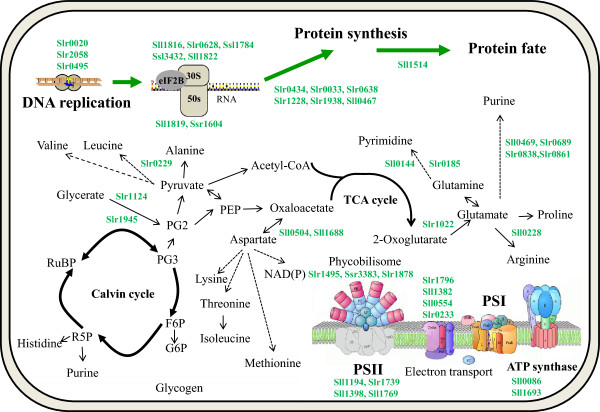
Schematic representation of central metabolic processes regulated by Slr1037.

Our previous transcriptomic and proteomic analyses of butanol response in *Synechocystis* unexpectedly revealed that a set of genes/proteins involved in the photosynthesis process were up-regulated [26,29]. Similar up-regulation of photosynthesis by another biofuel product, ethanol, was also found in *Synechocystis*, and confirmed by increased concentration of chlorophyll a concentration in cells [27], suggesting that the enhanced photosynthesis related activities were related to tolerance to butanol and ethanol in *Synechocystis*. Consistent with this discovery, we found that in the butanol-sensitive ∆*slr1037* mutant, many proteins involved in photosynthesis activity were down-regulated. Six proteins involved in photosystem and electron transfer, photosystem II 12 kDa extrinsic protein (Sll1194), photosystem II 13 kDa protein homolog (Slr1739), photosystem II reaction center 13 kDa protein (Sll1398), phycobilisome core component (Slr1459), phycobilisome small core linker polypeptide (Ssr3383), and cytochrome c553 (Sll1796), were down-regulated (Figure 3). Among them, Sll1194 and Sll1796 were found to be up-regulated by butanol, while Sll1398 was up-regulated by ethanol in the wild-type *Synechocystis*[26,27]. In addition, Slr0399 was also down-regulated in the ∆*slr1037* mutant. A previous study found that the deletion of *slr0399* has no significant phenotypic effects other than a decrease in thermotolerance under both photoautotrophic and photomixotrophic conditions, and suggested that Slr0399 is a chaperone-like protein that aids in, but is not essential for, quinone insertion and protein folding around Q(A) in photosystem II, and may also be involved in assembly of quinones in other photosynthetic and respiratory complexes [36].

Two proteins involved in the synthesis of phycobilisomes as antenna to enhance light harvesting for photosynthesis in cyanobacteria [37], phycocyanobilin lyase alpha subunit (Slr1878) and phycocyanin alpha phycocyanobilin lyase CpcE (Slr1098), were down-regulated in the butanol-sensitive ∆*slr1037* mutant. Moreover, ferredoxin (Sll1382) and ferredoxin:thioredoxin reductase (Sll0554) were also down-regulated. In cyanobacteria, ferredoxins are the main electron shuttles accepting electrons from photosystem I and delivering them to essential oxido-reductive pathways [38], while ferredoxin:thioredoxin reductase catalyzes the reduction of thioredoxins using the [Fe2S2] ferredoxin as a one-electron donor and as such plays a central role in light regulation of oxygenic photosynthesis [39]. Thioredoxins are small proteins involved in light-dependent enzyme regulation of many oxygen-evolving photosynthetic organisms from cyanobacteria to higher plants [40]. In the ∆*slr1037* mutant, thioredoxin M (Slr0233) was down-regulated. The protein has been found not essential for *Synechocystis* under normal photoautotrophic growth conditions, but the ∆*slr0233* mutant was slightly less tolerant to hydrogen peroxide compared to the wild type when grown photoautotrophically, and was suggested to have a role in the oxidative stress response in *Synechocystis*[40]. A probable thylakoid lumen protein Sll1769 was also down-regulated in the ∆*slr1037* mutant. As a previous study indicated that the cytochrome c550 needed to be transported into the thylakoid lumen and then contributed to optimal functional stability of photosystem II in *Synechocystis*[41], the thylakoid lumen protein could be important in maintaining proper photosynthesis.

Two proteins involved in glycolysis/gluconeogenesis, phosphoglycerate mutase (Slr1124) and bisphosphoglycerate-independent phosphoglycerate mutase (Slr1945), were down-regulated in the ∆*slr1037* mutant. The results were consistent with those of a previous study, which found an increased transcript concentration of phosphoglycerate mutase in cyanobacterium *S. elongatus* PCC 7942 during iron limitation [42], and our recent metabolomic analysis which showed increased 3-phosphoglycerate concentration in *Synechocystis* under butanol stress [29]. These results suggest that an increased withdrawal of metabolite flux from the Calvin cycle to increase the rate of glycolysis may represent an important mechanism in combating butanol toxicity. A protein essential for valine metabolism and degradation of leucine and isoleucine, 3-hydroxyisobutyrate dehydrogenase, was found down-regulated in the ∆*slr1037* mutant (Figure 3). In a recent study, 3-hydroxyisobutyrate dehydrogenase was shown to confer *Pseudomonas putida* and *E. coli* harboring the encoding gene *mmsB* improved tolerances to the organic solvent cyclohexane [43].

Compared with the wild-type strain, the ∆*slr1037* mutant grew poorly in BG11 supplemented with butanol (Figure 1). Consistent with a decreased growth pattern, proteins involved in core cellular metabolism, such as ATP synthesis, protein biosynthesis, protein fate, and biosynthesis of purines, pyrimidines, nucleosides and nucleotides, amino acids, and sucrose, were among the most down-regulated, although most of them are probably secondary responses of the *slr1037* knockout rather than under direct control by response regulator Slr1037 (Figure 3).

#### (*b*) Cellular processes

An oxidative stress response was observed in *E. coli* and *Synechocystis* treated with butanol [17,26]. Glutaredoxin (Ssr2061) and peptide methionine sulfoxide reductase (Sll1394) were down-regulated in the ∆*slr1037* mutant under butanol stress, suggesting that anti-oxidative responses are also regulated by the Slr1037 regulator (Table 2). Glutaredoxin has been implicated in maintenance of a normal cellular thiol/disufide ratio and the regeneration of oxidatively damaged proteins [44] and is essential for arsenate reduction in *Synechocystis*[45]. Peptide methionine sulfoxide reductase can catalyze oxidized methionine (methionine sulfoxides) back to methionines, providing cells with a mechanism to repair proteins damaged by reactive oxygen species rather than having them degraded and then re-synthesizing them *de novo*. Sll1394 was also up-regulated by ethanol exposure in the wild-type *Synechocystis*[27]. In addition, cyanoglobin (Slr2097), which has high oxygen affinity, was down-regulated in the ∆*slr1037* mutant under butanol stress. A previous study has indicated that cyanoglobin sequesters oxygen and presents it to, or is a part of, a terminal cytochrome oxidase complex in cyanobacteria [46]. One basic mechanism of salt acclimation in *Synechocystis* involves the accumulation of compatible solutes, such as glucosylglycerol [47]. The key enzyme in the production of the compatible solute glucosylglycerol, glucosylglycerol-phosphate synthase (Sll1566), was lower in the Δ*slr1037* mutant than in the wild type; however, the phenotypic analysis showed no change of salt tolerance in the Δ*slr1037* mutant (Figure 2). So far no information is available as to whether a similar mechanism against salt stress could be used against stress caused by butanol or other biofuel products.

**Table 2:** **Proteins down-regulated in the Δ** ***slr *** **mutant upon butanol exposure**

**Protein**	**Mutant_r1 versus wild-type_r1**		**Mutant_r2 versus wild-type_r1**		**Mutant_r1 versus wild-type_r2**		**Mutant_r2 versus wild-type_r2**		**Description**
	**Ratio (fold)**	**Sequence coverage (%)**	**Ratio (fold)**	**Sequence coverage (%)**	**Ratio (fold)**	**Sequence coverage (%)**	**Ratio (fold)**	**Sequence coverage (%)**	
Cell envelope									
Sll0274					-1.52	18.9			HglK-related
Sll1370					-1.78	8.7			Mannose-1-phosphate guanylyltransferase
Sll1483			-2.36	17.8					Periplasmic protein, similar to transforming growth factor induced protein
Slr0015							-1.541	31.7	Lipid-A-disaccharide synthase, putative
Slr0298					-1.75	13.8			FraH protein homolog
Slr0528	-1.601	21.3							UDP-N-acetylmuramyl-tripeptide synthetase
Slr0774					-1.79	21.2	-1.701	21.2	Protein-export membrane protein SecD
Slr0984							-1.88	115.3	CDP-glucose 4,6-dehydratase (EC 4.2.1.45) NAD + dependent
Slr1271					-1.94	11.8	-2.237	11.8	UDP-N-acetyl-D-mannosaminuronic acid transferase
Slr1510					-1.54	28.7			Fatty acid/phospholipid synthesis protein PlsX
Slr1975					-1.99	15.9	-1.54	15.9	N-acylglucosamine 2-epimerase
Slr0528	-1.601	21.3							UDP-N-acetylmuramyl-tripeptide synthetase
Cellular processes									
Sll0067			-1.79	10.9			-1.932	10.9	Glutathione S-transferase
Sll0708							-2.464	16.9	Dimethyladenosine transferase
Sll1394	-1.766	23.9			-1.76	23.9			Peptide methionine sulfoxide reductase
Sll1566					-1.61	13.8			Glucosylglycerol-phosphate synthase
Slr0399	-1.527	40.8							Chaperone-like protein for quinone binding in photosystem II
Slr0757			-2.16	25.7					Circadian clock protein KaiB homolog
Slr0758	-1.65	20.6							Circadian clock protein KaiC homolog
Slr0904	-1.525	10.4							Competence protein ComM homolog
Slr1829	-2.171	25.9	-1.52	25.9					Poly(3-hydroxyalkanoate) synthase
Slr2097			-1.82	58.6					Cyanoglobin
Ssr2061	-1.864	55.7							Glutaredoxin
Other functions									
Sll0368	-1.712	60.1	-1.65	60.1	-1.95	60.1	-1.817	60.1	Uracil phosphoribosyltransferase
Sll1020					-1.78	18.4			Probable glycosyltransferase
Sll1249			-1.65	11.7			-1.807	11.7	Pantothenate synthetase/cytidylate kinase
Sll1284					-1.69	31.9			Esterase
Slr2046					-2.01	1.5			MEGF1
Ssl2296							-1.761	77.1	Pterin-4a-carbinolamine dehydratase
Regulatory functions									
Sll1124							-1.809	2	Two-component sensor histidine kinase, phytochrome-like protein
Sll1672					-1.6	10.3			Two-component hybrid sensor and regulator
Sll2012			-1.6	9.4					Group-2 RNA polymerase sigma factor SigD
Slr0222					-1.75	4.8			Two-component hybrid sensor and regulator
Sll1124							-1.809	2	Two-component sensor histidine kinase, phytochrome-like protein
Transport and binding proteins									
Sll0064			-1.91	15.8	-1.96	15.8			Putative polar amino acid transport system
Sll0163							-1.643	5.7	WD-repeat protein
Sll0834					-2.36	11.7	-1.944	11.7	Low affinity sulfate transporter
Slr0559					-2.07	40.7			Branched chain amino acid ABC transporter
Slr0864					-1.51	10.8			ATP-binding protein of ABC transporter
Slr0925							-1.547	35.5	Single-stranded DNA-binding protein
Slr1149	-1.734	5							ATP-binding protein of ABC transporter
Slr1302							-1.786	6.1	Protein involved in constitutive low-affinity CO_2_ uptake
Slr1540	-1.642	18.6			-1.59	18.6			mRNA-binding protein
Slr1897					-1.54	18.9			Periplasmic sugar-binding protein of ABC transporter
Slr1974					-1.87	26.1			GTP binding protein
Sll0064			-1.91	15.8	-1.96	15.8			Putative polar amino acid transport system

Circadian rhythms of cyanobacteria are controlled by a cluster of three genes encoding circadian clock proteins (*kaiA*, *kaiB,* and *kaiC*), and the TCS systems involving a histidine kinase SasA and two response regulators, RpaA and RpaB, have been implicated in their regulation [48]. Proteomic analysis showed that KaiB (Slr0757) and KaiC (Slr0758) were both down-regulated in the ∆*slr1037* mutant under butanol stress (Table 2), implying that they are also regulated by the Slr1037 regulator. This result is consistent with our previous analysis showing that KaiB (Slr0757) was induced by ethanol in the wild-type *Synechocystis*[27]. The potential connection between circadian clock and biofuel stress in *Synechocystis* deserves further investigation.

#### (c) Cell envelope

Previous studies revealed that changes of cell membrane and envelope represent an important mechanism against biofuel toxicity in *Zymomonas mobilis*, *Saccharomyces cerevisiae*[49], and *Synechocystis*[26,27]. In the ∆*slr1037* mutant, 11 proteins related to cell membrane and envelope functions were down-regulated (Table 2). Interestingly, although involved in similar functions, such as membrane lipid biosynthesis, protein translocation, and formation of the extracellular matrix of the cell membrane, as those of proteins identified through analyzing butanol responses in the wild-type *Synechocystis*[26], down-regulated proteins identified in the ∆*slr1037* mutant represent a different set of responsive proteins, probably reflecting the complexity of the butanol tolerance network utilized by *Synechocystis*. However, it is worth noting that the HglK-related protein (Sll0274) and FraH protein homolog (Slr0298) were down-regulated in the ∆*slr1037* mutant. The HglK protein has been found necessary for localization of heterocyst-specific glycolipids in *Anabaena* sp. PCC 7120 [50], and the Fra protein influences filament integrity, diazotrophy, and localization of septal protein SepJ in the heterocyst-forming *Anabaena*[51]. The mechanism of these proteins against butanol toxicity in the unicellular *Synechocystis* may merit further investigation.

#### (d) Transport and binding proteins

Cross-membrane transporters for small molecules have been suggested as one important factor against biofuel toxicity in various microbes [40,52]. Our previous proteomic analysis also identified nine putative transporters with induced expression level by butanol [26]. In the ∆*slr1037* mutant, eight proteins related to transport and binding protein functions were down-regulated (Table 2), including a branched chain amino acid ABC transporter (Slr0559), a periplasmic amino acid-binding protein (Sll0064), a putative polar amino acid transport system (Sll0834), a low-affinity sulfate transporter, a periplasmic sugar-binding protein of ABC transporter (Slr1897), and two ATP-binding proteins of ABC transporters (Slr0864, Slr1149). Currently, there is no information available regarding substrate specificity of these proteins in *Synechocystis*, and their exact physiological function in butanol tolerance still needs further investigation. Two systems have been identified in *Synechocystis* to achieve CO_2_-concentrating and active CO_2_ uptake, one inducible at low CO_2_ conditions and another constitutive [53]. Our analysis showed that Slr1302, a protein involved in constitutive low-affinity CO_2_ uptake, was down-regulated in the ∆*slr1037* mutant (Table 2). Similar regulation of two bicarbonate transporters (Slr0040 and Slr1512) was also found in *Synechocystis* treated with ethanol [27]. In addition, a WD-repeat protein (Sll0163) was down-regulated in the ∆*slr1037* mutant (Table 2). *Synechocystis* possesses several open reading frames encoding putative WD-repeat proteins, one of which, the Hat protein, has been found to be involved in the control of a high-affinity transport system for inorganic carbon when the cells are grown under a limiting concentration of this carbon substrate [54]. Although the ∆*slr1037* mutant was growing poorly under butanol stress, no protein involved in the CO_2_ fixation pathway was differentially regulated, consistent with the constant expression of the pathway in the wild-type *Synechocystis* treated with butanol [26]; however, the results are in contrast with the deceased expression of CO_2_ fixation pathway under stress conditions, such as sulfur starvation or low phosphate [55,56], implying that it may be CO_2_ transport rather than CO_2_ fixation that is involved in butanol tolerance mechanisms in *Synechocystis*.

#### (e) Regulatory proteins

Four regulatory proteins were down-regulated in the ∆*slr1037* mutant (Table 2), including a phytochrome-like sensor histidine kinase (Sll1124), two hybrid-type sensor and regulator proteins (Sll1672, Slr0222), and an RNA polymerase sigma factor SigD (Sll2012). As *slr1037* is an orphan response regulator gene, we expected that its cognate histidine kinase gene may be standalone and affected in the ∆*slr1037* mutant. Examination of three differentially regulated TCS genes showed that Sll1124 is an orphan histidine kinase necessary for growth under blue light [57], Slr0222 is also an orphan functionally unknown histidine kinase, and *sll1672* is clustered with a downstream response regulator Rre2 (*sll1673*). Although group 2 sigma factor SigD is nonessential in *Synechocystis*, it was involved in acclimation to salt- or sorbitol-induced osmotic stresses [58].

#### (f) Hypothetical proteins

Sixty-eight hypothetical proteins were down-regulated in the ∆*slr1037* mutant (Additional file 3: Table S2). Notably, Sll0167 and Slr1403 were down-regulated in all four biological replicates. In addition, several groups of hypothetical proteins with encoding genes clustered together in the chromosome were all down-regulated by the gene knockout, including Slr1001-Slr1002, Slr1259-Slr1260-Slr1261, and Slr1275-Slr1276. Using RT-qPCR, we also confirmed that *slr1403* and *slr126*0 were down-regulated at transcriptional level (Table 1). Although currently no functional information is available for these hypothetical proteins, their similar change patterns suggested possible involvement in butanol tolerance and may be worthy of further investigation.

### Identification of possible Slr1037 binding sites

**Figure 4:**
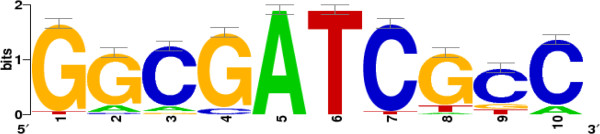
**Putative regulatory module identified upstream of the genes encoding ∆*slr1037*-responsive proteins.** The motif is represented by a sequence logo generated by the WebLogo software [62].

To further define the possible regulatory network mediated by Slr1037, we conducted a promoter DNA-binding motif search using 500 bp sequences extracted from the upstream regions of all the genes encoding the down-regulated proteins in the ∆*slr1037* mutant using the Gibbs Motif Sampler software [59,60]. This analysis showed that the top conserved motif identified was a palindrome containing 10 total sites with the DNA sequence “GGCGATCGCC” (Figure 4) [61,62]. The motif has several conserved positions with information bits greater than 0.5 [60]. The genes associated with the first motif included *sll0067* encoding a putative glutathione S-transferase, *ssr2061* encoding a glutaredoxin 3 (*grx3*), and *slr1149* encoding an ABC transporter, which have been suggested as being related to stress responses in cyanobacteria [44,45,63,64]. As a preliminary prediction, the functionality of the motif associated with the Slr1037 response regulator will need more experimental proof.

## Conclusions

Increasing studies have found that tolerance to biofuels may involve responses of multiple cellular functions [14,15]; thus, it may be challenging to achieve biofuel tolerance by engineering a single metabolic gene, enzyme, or even a pathway. Alternatively, the manipulation of regulatory genes could provide a route to complex phenotypes, since the regulatory genes tend to control a set of genes related to certain functions [18]. To improve biofuel tolerance in the photosynthetic cyanobacterial hosts that can be used to produce carbon-neutral products directly from CO_2_, in this study, we conducted experiments to identify regulatory genes related to butanol tolerance in *Synechocystis*. The efforts allowed identification of *slr1037* encoding an orphan response regulator that was involved in butanol tolerance in *Synechocystis*. Using a quantitative iTRAQ LC-MS/MS proteomics approach coupled with quantitative RT-qPCR, we further determined the possible regulatory network mediated by Slr1037. The results showed that along with the *slr1037* deletion, proteins involved in photosynthesis and glycolysis/gluconeogenesis of central metabolic processes, and glutaredoxin, peptide methionine sulfoxide reductase, and glucosylglycerol-phosphate synthase with stress-responsive functions were down-regulated, suggesting that Slr1037 exhibited regulation to a wide range of cellular functions in combating butanol stress. Finally, it should be noted that, in order to cover all possible targets regulated by Slr1037, we used a relatively low criterion in determining responsive proteins, and the proteins differentially regulated in any of the replicates were reported. Thus, more work is still needed to further validate whether these molecular targets are directly controlled by the Slr1037 response regulator. Nevertheless, as the first regulatory protein discovered so far that was related to butanol tolerance in *Synechocystis*, the *slr1037* gene can serve as an important target for engineering transcriptional machinery in order to improve butanol tolerance and productivity in photosynthetic *Synechocystis*.

## Methods

### Bacterial growth conditions

*Synechocystis* sp. PCC 6803 and gene knockout mutants were grown in BG11 medium (pH 7.5) under a light intensity of approximately 50 μmol photons m^-2^ s^-1^ in an illuminating incubator of 130 rpm at 30°C (HNY-211B Illuminating Shaker, Honour, China) [26,27]. Cell density was measured at OD_630_ on an ELx808 Absorbance Microplate Reader (BioTek, Winooski, VT, USA). For growth and butanol treatment, 10 mL fresh cells at OD_630_ of 0.2 were collected by centrifugation and then inoculated into 50 mL of BG11 liquid medium in a 250-mL flask. Butanol of analytical purity was purchased from Merck (Whitehouse Station, NJ, USA). The growth experiments were repeated at least three times to confirm the growth patterns. Cells for proteomics analysis were collected by centrifugation at 8,000 × *g* for 10 min at 4°C.

### Construction and analysis of knockout mutants

A fusion PCR-based method was employed for the construction of gene knockout fragments [65]. Briefly, for the gene target selected, three sets of primers were designed to amplify a linear DNA fragment containing the chloramphenicol resistance cassette (amplified from a plasmid pACYC184) with two flanking arms of DNA upstream and downstream of the target gene. The linear fused PCR amplicon was used directly for transformation into *Synechocystis* by natural transformation. The chloramphenicol-resistant transformants were obtained and passed several times on fresh BG11 plates supplemented with 10 μg/mL chloramphenicol to achieve complete chromosome segregation (confirmed by PCR). Two TCSTS genes, *sll1708* and *slr1037,* and one transcriptional regulator gene, *sll0690,* that were previously found differentially regulated by butanol exposure [26,29], were selected for the construction of gene knockout mutants. The successful knockout mutants were confirmed by PCR and sequencing analysis. The comparative growth analysis of the wild-type *Synechocystis* and the mutants was performed in 100-mL flasks each with 10 mL BG11 medium with or without 0.25% (*v*/*v*) butanol. The cultivation conditions were the same as described above. Growth analysis was performed in biological triplicates.

### Proteomics analysis

#### i) Protein preparation and digestion

For each sample, 10 mg of cells were frozen by liquid nitrogen immediately after centrifugation and washed with phosphate buffer (pH 7.2). The cells were broken with sonication cracking at low temperature. The cell pellets were then resuspended in a lysis buffer (8 M urea, 4% CHAPS, 40 mM Tris-HCl) with 1 mM PMSF and 2 mM ethylenediaminetetraacetic acid (EDTA) (final concentration). After 5 min of vigorous vortexing, dithiothreitol (DTT) was also added to a final concentration of 10 mM. After mixing, the samples were centrifuged for 20 min at 20,000 × *g*, and the supernatant was mixed well with ice-cold acetone (1:4, *v*/*v*) with 30 mM DTT. After repeating this step twice, the supernatants were combined and precipitated at -20°C overnight, and stored at -80°C prior to sample cleanup if not for immediate use. For digestion, protein pellets from the previous step were resuspended in digestion buffer (100 mM triethylammonium bicarbonate, TEAB, 0.05% *w***/***v* sodium dodecyl sulfate, SDS) to a final concentration of 1 mg/mL (total protein measured by bicinchoninic acid assay (Sigma, St. Louis, MO, USA)). Equal aliquots (500 μg) from each lysate were then digested with trypsin overnight at 37°C (Sigma; 1:40 *w*/*w* added at 0 and 2 h) and lyophilized.

#### ii) iTRAQ labeling

The iTRAQ labeling of peptide samples derived from the control and butanol-treated samples was performed using the iTRAQ Reagent 8-plex kit (Applied Biosystems, Foster City, CA) according to the manufacturer’s protocol. Four samples (two biological replicates for the wild-type control and two biological replicates of the ∆*slr1037* mutant, respectively) were iTRAQ labeled. The peptides were labeled with respective isobaric tags, incubated for 2 h, and vacuum centrifuged to dryness. The labeled wild-type control and ∆*slr1037* mutant replicate samples were 1:1 pooled, and generated four combinations of samples, which were reconstituted in buffer A (10 mM KH_2_PO_4_, 25% acetonitrile, pH 2.85). The iTRAQ labeled peptides were fractionated using a PolySULFOETHYL ATM SCX column (200 × 4.6 mm, 5 μm particle size, 200 Aº pore size) by an HPLC system (Shimadzu, Japan) at flow rate 1.0 mL min^-1^. The 50-min HPLC gradient consisted of 100% buffer A (10 mM KH_2_PO_4_, 25% acetonitrile, pH 2.85) for 5 min; 0 to 20% buffer B (10 mM KH_2_PO_4_, 25% ACN, 500 mM KCL, pH 3.0) for 15 min; 20 to 40% buffer B for 10 min; 40 to 100% buffer B for 5 min followed by 100% buffer A for 10 min. The chromatograms were recorded at 218 nm. The collected fractions were desalted with Sep-Pak® Vac C18 cartridges (Waters, Milford, MA), concentrated to dryness using a vacuum centrifuge, and reconstituted in 0.1% formic acid for LC-MS/MS analysis.

#### iii) LC-MS/MS proteomic analysis

The mass spectroscopy analysis was performed using an AB SCIEX TripleTOF™ 5600 mass spectrometer (AB SCIEX, Framingham, MA, USA), coupled with an online micro flow HPLC system (Shimadzu, Japan) as described before [26,27]. The peptides were separated using a Nanobore C18 column with a PicoFrit nanospray tip (75 μm ID × 15 cm, 5 μm particles) (New Objectives, Woburn, MA). The separation was performed at a constant flow rate of 20 μL min^-1^, with a splitter to get an effective flow rate of 0.2 μL min^-1^. The mass spectrometer data was acquired in the positive ion mode, with a selected mass range of 300 to 2,000 m/z. Peptides with +2 to +4 charge states were selected for MS/MS. The three most abundantly charged peptides above a count threshold were selected for MS/MS and dynamically excluded for 30 s with ±30 mDa mass tolerance. Smart information-dependent acquisition (IDA) was activated with automatic collision energy and automatic MS/MS accumulation. The fragment intensity multiplier was set to 20, and the maximum accumulation time was 2 s. The peak areas of the iTRAQ reporter ions reflect the relative abundance of the proteins in the samples. For peptide identification, the Triple TOF 5600 mass spectrometer used in this study has high mass accuracy (less than 2 ppm). Other identification parameters used included: fragment mass tolerance: ± 0.1 Da; mass values: monoisotopic; variable modifications: Gln- > pyro-Glu (N-term Q), oxidation (M), iTRAQ8plex (Y); peptide mass tolerance: 0.05 Da; max missed cleavages: 1; fixed modifications: carbamidomethyl (C), iTRAQ8plex (N-term), iTRAQ8plex (K); other parameters: default.

#### iv) Proteomic data analysis

The MS data were processed using Proteome Discoverer software (Version 1.2.0.208) (Thermo Scientific) to generate the peak list. The default parameters of Proteome Discoverer software (Version 1.2.0.208) were used. The data acquisition was performed with Analyst QS 2.0 software (Applied Biosystems/MDS SCIEX). Protein identification and quantification were performed using Mascot 2.3.02 (Matrix Science, London, UK) [26,27]. For iTRAQ quantification, the peptide for quantification was automatically selected by the algorithm to calculate the reporter peak area, error factor (EF), and *p*-value (default parameters in the Mascot software package). The resulting data set was auto bias-corrected to eliminate any variations imparted due to the unequal mixing during combining of the different labeled samples. The genome sequence and annotation information of *Synechocystis* sp. PCC 6803 were downloaded from NCBI, the Comprehensive Microbial Resource (CMR) of TIGR, and CyanoBase [35]. Proteins with a 1.5 fold or more change between the ∆*slr1037* mutant and the wild-type control samples and a *p*-value of statistical evaluation less than 0.05 were determined as differentially expressed proteins. The quantitation was performed at the peptide level by following the procedures described previously [26,27]. The Student’s t-test was performed using the Mascot 2.3.02 software.

### Quantitative real-time reverse tanscription-PCR analysis

The identical cell samples used for proteomics analysis were used for RT-qPCR analysis. cDNAs were synthesized using RevertAidTM Reverse Transcriptase (Fermentas, Glen Burnie, MD, USA). The qPCR reaction was carried out in 20 μL reactions containing 10 μL of SYBR® Green PCR Master Mix (Applied Biosystems, Foster City, CA), and 2 μL of each PCR primer at 2 mM, employing the StepOnePlus™ Real-Time PCR System (Applied Biosystems, Foster City, CA), under the following conditions: 50°C for 2 min and 95°C for 10 min, followed by 40 cycles of 95°C for 15 s and 60°C for 1 min. Quantification of gene expression was determined according to the standard process of RT-qPCR, which used serial dilutions of known concentrations of chromosome DNA as a template to make a standard curve. The *rnpB* gene (*6803 s01*) encoding RNase P subunit B was used as an internal control [66]. Three technical replicates were performed for each gene. Data analysis was carried out using the StepOnePlus analytical software (Applied Biosystems, Foster City, CA). The data was presented as ratios of the amount of normalized transcript in the mutant to that from the wild-type control. The gene ID and their related primer sequences used for real-time RT-qPCR analysis are listed in Additional file 1: Table S1.

### Promoter analysis and motif identification

The Gibbs Motif Sampler software from the Biometrics Laboratory of Wadsworth Center, Albany, NY, USA was used to identify matrix models describing DNA sequence motifs present upstream of genes responsive to butanol treatment [59,60]. Regions representing approximately 500 base pairs of the DNA sequences upstream of the translational start site of responsive genes to butanol stress were extracted from the NCBI genome database using the Regulatory Sequence Analysis Tools (RSAT) [61]. Both strands of each sequence were searched and possible motif locations were identified using the motif matrix score obtained from the Gibbs Motif Sampler software. The multilevel consensus sequence for each motif was then used to generate a sequence logo that is a graphical representation of nucleic acid multiple sequence alignment [62].

## Abbreviations

iTRAQ: isobaric tag for relative and absolute quantitation; LC-MS/MS: liquid chromatography-tandem mass spectrometry; RT-PCR: reverse-transcription PCR; TCSTS: two-component signal transduction system.

## Competing interests

The authors declare that they have no competing interests.

## Authors’ contributions

LC, JW, and WZ conceived of the study. LC, JW, and WZ drafted the manuscript. LW and LC carried out the RT-PCR, mutant construction, phenotypic analysis, and proteomic analysis. All authors read and approved the final manuscript.

## Supplementary Material

Additional file 1: Table S1 Primers used in this study. Click here for file

Additional file 2: Figure S1 Repeatability between biological replicates. Wild-type **(A)** and ∆*slr1037* mutant **(B)**, respectively. Distribution of iTRAQ log ratios of the detected proteins between ∆*slr1037* biological replicate 1 and the wild type **(C)** and between ∆*slr1037* biological replicate 2 and the wild type** (D)**. Click here for file

Additional file 3: Table S2 Hypothetical proteins differentially regulated in the ∆*slr1037* mutant. Click here for file
