# Supplementary material for: Butanol tolerance regulated by a two-component response regulator Slr1037 in photosynthetic Synechocystis sp. PCC 6803
Source: Biotechnol Biofuels. 2014 Jun 11;7:89. doi: 10.1186/1754-6834-7-89 (PMC4057619; doi:10.1186/1754-6834-7-89)
Supplement: Additional file 2: Figure S1 — Repeatability between biological replicates. Wild-type (A) and ∆slr1037 mutant (B), respectively. Distribution of iTRAQ log ratios of the detected proteins between ∆slr1037 biological replicate 1 and the wild type (C) and between ∆slr1037 biological replicate 2 and the wild type (D). [file 1754-6834-7-89-S2.ppt]

## Slide 1
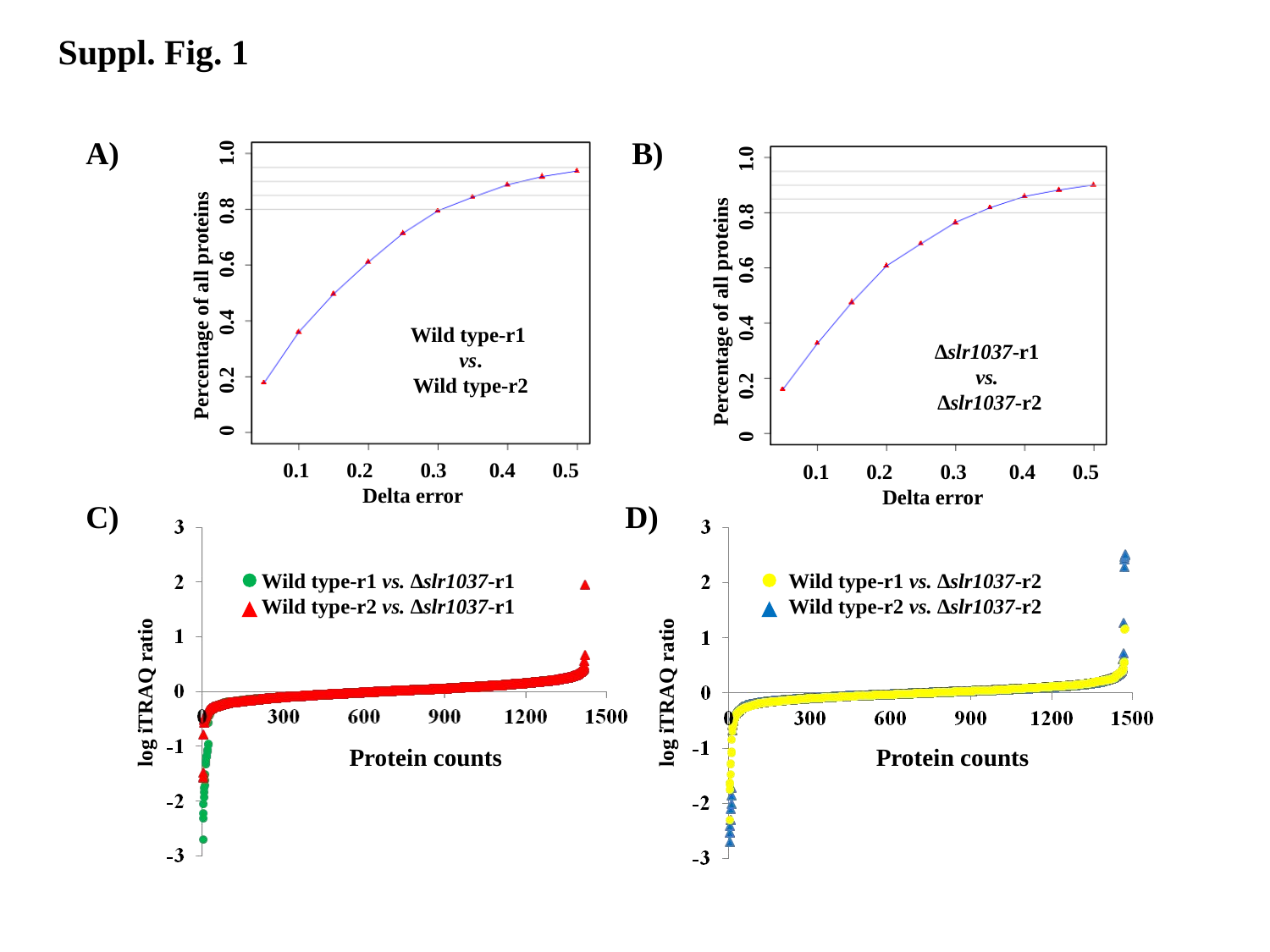

Suppl. Fig. 1
 B)
C) D)
 Percentage of all proteins
0 0.2 0.4 0.6 0.8 1.0
 Percentage of all proteins
0 0.2 0.4 0.6 0.8 1.0
Wild type-r1
vs.
Wild type-r2
∆slr1037-r1
vs.
∆slr1037-r2
 0.1 0.2 0.3 0.4 0.5
 Delta error
 0.1 0.2 0.3 0.4 0.5
 Delta error
Wild type-r1 vs. ∆slr1037-r1
Wild type-r2 vs. ∆slr1037-r1
Wild type-r1 vs. ∆slr1037-r2
Wild type-r2 vs. ∆slr1037-r2
log iTRAQ ratio
log iTRAQ ratio
Protein counts
Protein counts
